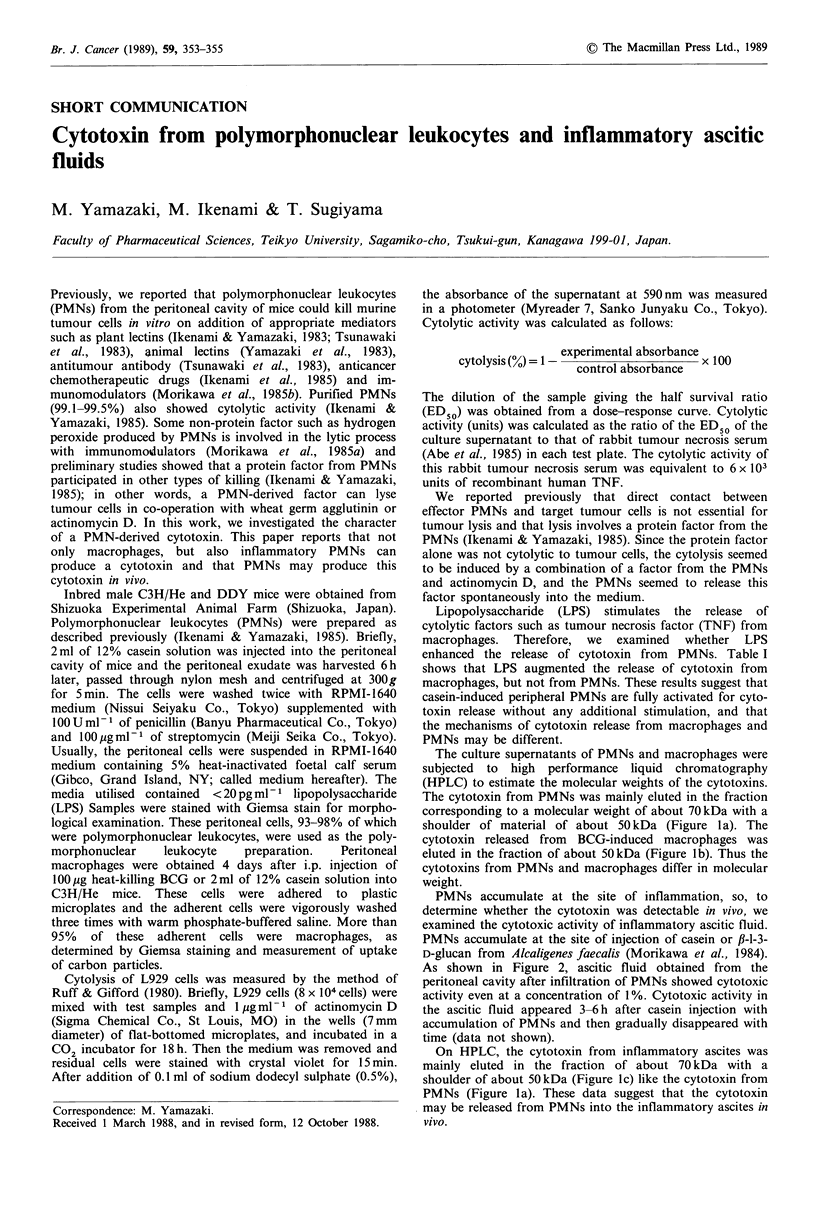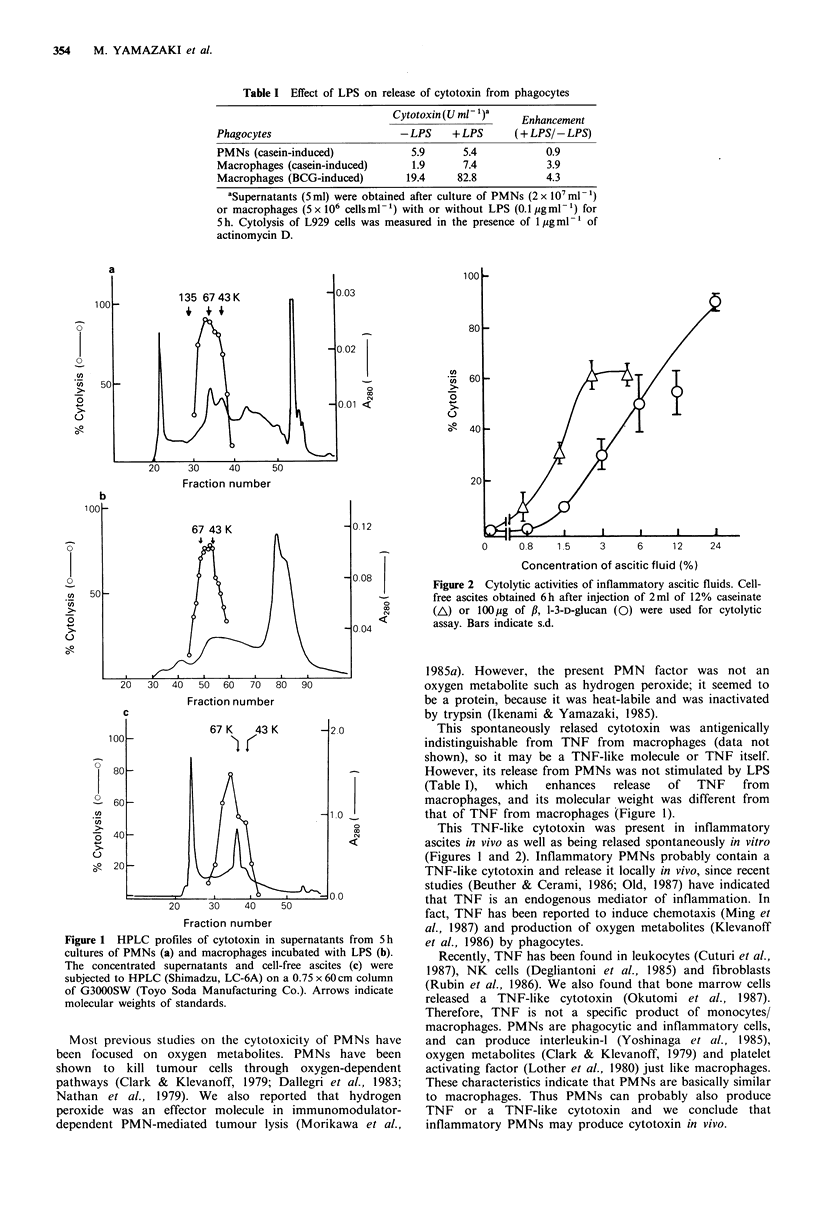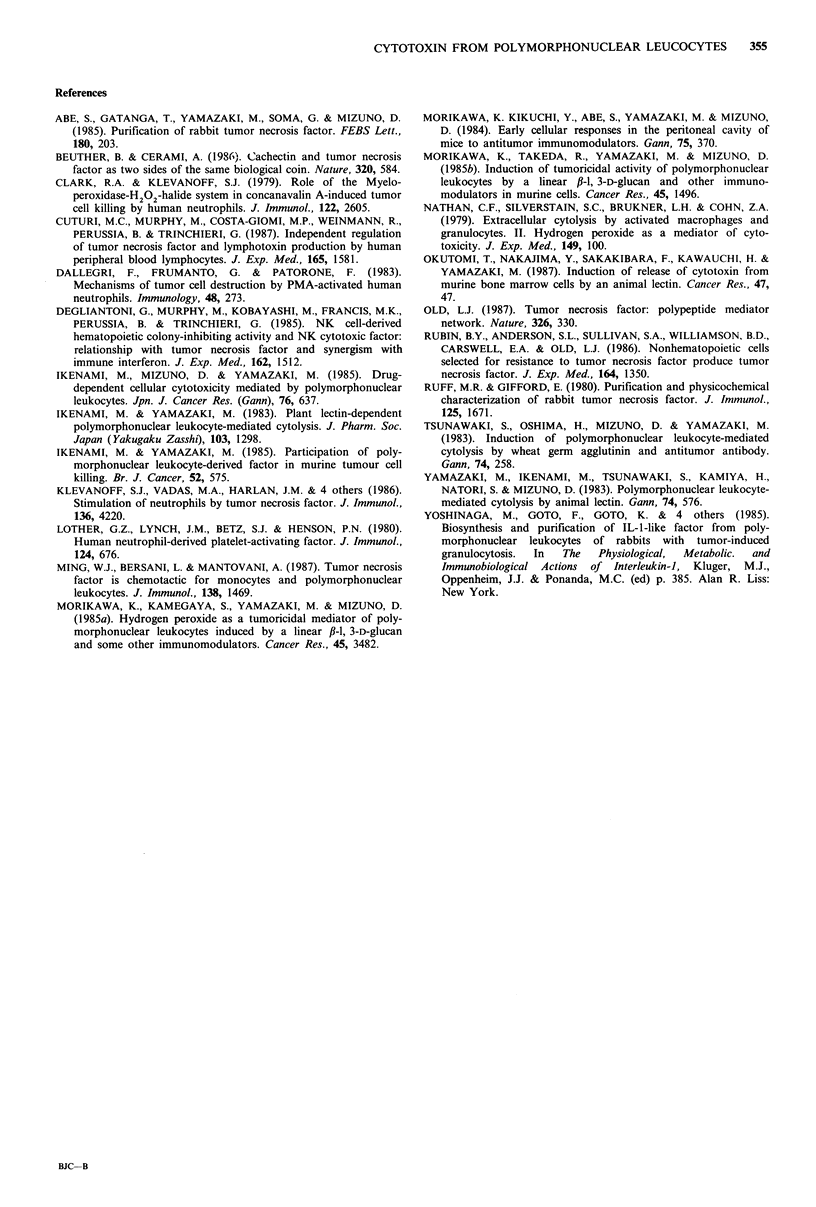# Cytotoxin from polymorphonuclear leukocytes and inflammatory ascitic fluids.

**DOI:** 10.1038/bjc.1989.70

**Published:** 1989-03

**Authors:** M. Yamazaki, M. Ikenami, T. Sugiyama

**Affiliations:** Faculty of Pharmaceutical Sciences, Teikyo University, Kanagawa, Japan.


*Br. J. Cancer* (1989), **59**, 353–355
© The Macmillan Press Ltd., 1989

SHORT COMMUNICATION

### Cytotoxin from polymorphonuclear leukocytes and inflammatory ascitic fluids

M. Yamazaki, M. Ikenami & T. Sugiyama

*Faculty of Pharmaceutical Sciences, Teikyo University, Sagamiko-cho, Tsukui-gun, Kanagawa 199-01, Japan.*

Previously, we reported that polymorphonuclear leukocytes (PMNs) from the peritoneal cavity of mice could kill murine tumour cells *in vitro* on addition of appropriate mediators such as plant lectins (Ikenami & Yamazaki, 1983; Tsunawaki *et al.*, 1983), animal lectins (Yamazaki *et al.*, 1983), antitumour antibody (Tsunawaki *et al.*, 1983), anticancer chemotherapeutic drugs (Ikenami *et al.*, 1985) and immunomodulators (Morikawa *et al.*, 1985*b*). Purified PMNs (99.1–99.5%) also showed cytolytic activity (Ikenami & Yamazaki, 1985). Some non-protein factor such as hydrogen peroxide produced by PMNs is involved in the lytic process with immunomodulators (Morikawa *et al.*, 1985*a*) and preliminary studies showed that a protein factor from PMNs participated in other types of killing (Ikenami & Yamazaki, 1985); in other words, a PMN-derived factor can lyse tumour cells in co-operation with wheat germ agglutinin or actinomycin D. In this work, we investigated the character of a PMN-derived cytotoxin. This paper reports that not only macrophages, but also inflammatory PMNs can produce a cytotoxin and that PMNs may produce this cytotoxin *in vivo*.

Inbred male C3H/He and DDY mice were obtained from Shizuoka Experimental Animal Farm (Shizuoka, Japan). Polymorphonuclear leukocytes (PMNs) were prepared as described previously (Ikenami & Yamazaki, 1985). Briefly, 2 ml of 12% casein solution was injected into the peritoneal cavity of mice and the peritoneal exudate was harvested 6 h later, passed through nylon mesh and centrifuged at 300 *g* for 5 min. The cells were washed twice with RPMI-1640 medium (Nissui Seiyaku Co., Tokyo) supplemented with 100 U ml$^{-1}$ of penicillin (Banyu Pharmaceutical Co., Tokyo) and 100 $\mu$g ml$^{-1}$ of streptomycin (Meiji Seika Co., Tokyo). Usually, the peritoneal cells were suspended in RPMI-1640 medium containing 5% heat-inactivated foetal calf serum (Gibco, Grand Island, NY; called medium hereafter). The media utilised contained $<20$ pg ml$^{-1}$ lipopolysaccharide (LPS) Samples were stained with Giemsa stain for morphological examination. These peritoneal cells, 93–98% of which were polymorphonuclear leukocytes, were used as the polymorphonuclear leukocyte preparation. Peritoneal macrophages were obtained 4 days after i.p. injection of 100 $\mu$g heat-killing BCG or 2 ml of 12% casein solution into C3H/He mice. These cells were adhered to plastic microplates and the adherent cells were vigorously washed three times with warm phosphate-buffered saline. More than 95% of these adherent cells were macrophages, as determined by Giemsa staining and measurement of uptake of carbon particles.

Cytolysis of L929 cells was measured by the method of Ruff & Gifford (1980). Briefly, L929 cells ($8 \times 10^4$ cells) were mixed with test samples and 1 $\mu$g ml$^{-1}$ of actinomycin D (Sigma Chemical Co., St Louis, MO) in the wells (7 mm diameter) of flat-bottomed microplates, and incubated in a $CO_2$ incubator for 18 h. Then the medium was removed and residual cells were stained with crystal violet for 15 min. After addition of 0.1 ml of sodium dodecyl sulphate (0.5%), the absorbance of the supernatant at 590 nm was measured in a photometer (Myreader 7, Sanko Junyaku Co., Tokyo). Cytolytic activity was calculated as follows:

$$\text{cytolysis}\,(\%) = 1 - \frac{\text{experimental absorbance}}{\text{control absorbance}} \times 100$$

The dilution of the sample giving the half survival ratio ($ED_{50}$) was obtained from a dose–response curve. Cytolytic activity (units) was calculated as the ratio of the $ED_{50}$ of the culture supernatant to that of rabbit tumour necrosis serum (Abe *et al.*, 1985) in each test plate. The cytolytic activity of this rabbit tumour necrosis serum was equivalent to $6 \times 10^3$ units of recombinant human TNF.

We reported previously that direct contact between effector PMNs and target tumour cells is not essential for tumour lysis and that lysis involves a protein factor from the PMNs (Ikenami & Yamazaki, 1985). Since the protein factor alone was not cytolytic to tumour cells, the cytolysis seemed to be induced by a combination of a factor from the PMNs and actinomycin D, and the PMNs seemed to release this factor spontaneously into the medium.

Lipopolysaccharide (LPS) stimulates the release of cytolytic factors such as tumour necrosis factor (TNF) from macrophages. Therefore, we examined whether LPS enhanced the release of cytotoxin from PMNs. Table I shows that LPS augmented the release of cytotoxin from macrophages, but not from PMNs. These results suggest that casein-induced peripheral PMNs are fully activated for cytotoxin release without any additional stimulation, and that the mechanisms of cytotoxin release from macrophages and PMNs may be different.

The culture supernatants of PMNs and macrophages were subjected to high performance liquid chromatography (HPLC) to estimate the molecular weights of the cytotoxins. The cytotoxin from PMNs was mainly eluted in the fraction corresponding to a molecular weight of about 70 kDa with a shoulder of material of about 50 kDa (Figure 1a). The cytotoxin released from BCG-induced macrophages was eluted in the fraction of about 50 kDa (Figure 1b). Thus the cytotoxins from PMNs and macrophages differ in molecular weight.

PMNs accumulate at the site of inflammation, so, to determine whether the cytotoxin was detectable *in vivo*, we examined the cytotoxic activity of inflammatory ascitic fluid. PMNs accumulate at the site of injection of casein or $\beta$-1-3-D-glucan from *Alcaligenes faecalis* (Morikawa *et al.*, 1984). As shown in Figure 2, ascitic fluid obtained from the peritoneal cavity after infiltration of PMNs showed cytotoxic activity even at a concentration of 1%. Cytotoxic activity in the ascitic fluid appeared 3–6 h after casein injection with accumulation of PMNs and then gradually disappeared with time (data not shown).

On HPLC, the cytotoxin from inflammatory ascites was mainly eluted in the fraction of about 70 kDa with a shoulder of about 50 kDa (Figure 1c) like the cytotoxin from PMNs (Figure 1a). These data suggest that the cytotoxin may be released from PMNs into the inflammatory ascites *in vivo*.

Correspondence: M. Yamazaki.
Received 1 March 1988, and in revised form, 12 October 1988.

**Table I** Effect of LPS on release of cytotoxin from phagocytes

| *Phagocytes* | *Cytotoxin* $(U\ ml^{-1})^a$ $-LPS$ | $+LPS$ | *Enhancement* $(+LPS/-LPS)$ |
|---|---|---|---|
| PMNs (casein-induced) | 5.9 | 5.4 | 0.9 |
| Macrophages (casein-induced) | 1.9 | 7.4 | 3.9 |
| Macrophages (BCG-induced) | 19.4 | 82.8 | 4.3 |

[a]Supernatants (5 ml) were obtained after culture of PMNs ($2\times10^7\ ml^{-1}$) or macrophages ($5\times10^6$ cells $ml^{-1}$) with or without LPS (0.1 $\mu g\ ml^{-1}$) for 5 h. Cytolysis of L929 cells was measured in the presence of 1 $\mu g\ ml^{-1}$ of actinomycin D.

**Figure 1** HPLC profiles of cytotoxin in supernatants from 5 h cultures of PMNs (**a**) and macrophages incubated with LPS (**b**). The concentrated supernatants and cell-free ascites (**c**) were subjected to HPLC (Shimadzu, LC-6A) on a 0.75 × 60 cm column of G3000SW (Toyo Soda Manufacturing Co.). Arrows indicate molecular weights of standards.

**Figure 2** Cytolytic activities of inflammatory ascitic fluids. Cell-free ascites obtained 6 h after injection of 2 ml of 12% caseinate (△) or 100 $\mu g$ of $\beta$, 1-3-D-glucan (○) were used for cytolytic assay. Bars indicate s.d.

Most previous studies on the cytotoxicity of PMNs have been focused on oxygen metabolites. PMNs have been shown to kill tumour cells through oxygen-dependent pathways (Clark & Klevanoff, 1979; Dallegri *et al.*, 1983; Nathan *et al.*, 1979). We also reported that hydrogen peroxide was an effector molecule in immunomodulator-dependent PMN-mediated tumour lysis (Morikawa *et al.*, 1985*a*). However, the present PMN factor was not an oxygen metabolite such as hydrogen peroxide; it seemed to be a protein, because it was heat-labile and was inactivated by trypsin (Ikenami & Yamazaki, 1985).

This spontaneously relased cytotoxin was antigenically indistinguishable from TNF from macrophages (data not shown), so it may be a TNF-like molecule or TNF itself. However, its release from PMNs was not stimulated by LPS (Table I), which enhances release of TNF from macrophages, and its molecular weight was different from that of TNF from macrophages (Figure 1).

This TNF-like cytotoxin was present in inflammatory ascites *in vivo* as well as being relased spontaneously *in vitro* (Figures 1 and 2). Inflammatory PMNs probably contain a TNF-like cytotoxin and release it locally *in vivo*, since recent studies (Beuther & Cerami, 1986; Old, 1987) have indicated that TNF is an endogenous mediator of inflammation. In fact, TNF has been reported to induce chemotaxis (Ming *et al.*, 1987) and production of oxygen metabolites (Klevanoff *et al.*, 1986) by phagocytes.

Recently, TNF has been found in leukocytes (Cuturi *et al.*, 1987), NK cells (Degliantoni *et al.*, 1985) and fibroblasts (Rubin *et al.*, 1986). We also found that bone marrow cells released a TNF-like cytotoxin (Okutomi *et al.*, 1987). Therefore, TNF is not a specific product of monocytes/macrophages. PMNs are phagocytic and inflammatory cells, and can produce interleukin-1 (Yoshinaga *et al.*, 1985), oxygen metabolites (Clark & Klevanoff, 1979) and platelet activating factor (Lother *et al.*, 1980) just like macrophages. These characteristics indicate that PMNs are basically similar to macrophages. Thus PMNs can probably also produce TNF or a TNF-like cytotoxin and we conclude that inflammatory PMNs may produce cytotoxin *in vivo*.

**References**

ABE, S., GATANGA, T., YAMAZAKI, M., SOMA, G. & MIZUNO, D. (1985). Purification of rabbit tumor necrosis factor. *FEBS Lett.*, **180**, 203.

BEUTHER, B. & CERAMI, A. (1986). Cachectin and tumor necrosis factor as two sides of the same biological coin. *Nature*, **320**, 584.

CLARK, R.A. & KLEVANOFF, S.J. (1979). Role of the Myeloperoxidase-$H_2O_2$-halide system in concanavalin A-induced tumor cell killing by human neutrophils. *J. Immunol.*, **122**, 2605.

CUTURI, M.C., MURPHY, M., COSTA-GIOMI, M.P., WEINMANN, R., PERUSSIA, B. & TRINCHIERI, G. (1987). Independent regulation of tumor necrosis factor and lymphotoxin production by human peripheral blood lymphocytes. *J. Exp. Med.*, **165**, 1581.

DALLEGRI, F., FRUMANTO, G. & PATORONE, F. (1983). Mechanisms of tumor cell destruction by PMA-activated human neutrophils. *Immunology*, **48**, 273.

DEGLIANTONI, G., MURPHY, M., KOBAYASHI, M., FRANCIS, M.K., PERUSSIA, B. & TRINCHIERI, G. (1985). NK cell-derived hematopoietic colony-inhibiting activity and NK cytotoxic factor: relationship with tumor necrosis factor and synergism with immune interferon. *J. Exp. Med.*, **162**, 1512.

IKENAMI, M., MIZUNO, D. & YAMAZAKI, M. (1985). Drug-dependent cellular cytotoxicity mediated by polymorphonuclear leukocytes. *Jpn. J. Cancer Res.* (*Gann*), **76**, 637.

IKENAMI, M. & YAMAZAKI, M. (1983). Plant lectin-dependent polymorphonuclear leukocyte-mediated cytolysis. *J. Pharm. Soc. Japan* (*Yakugaku Zasshi*), **103**, 1298.

IKENAMI, M. & YAMAZAKI, M. (1985). Participation of polymorphonuclear leukocyte-derived factor in murine tumour cell killing. *Br. J. Cancer*, **52**, 575.

KLEVANOFF, S.J., VADAS, M.A., HARLAN, J.M. & 4 others (1986). Stimulation of neutrophils by tumor necrosis factor. *J. Immunol.*, **136**, 4220.

LOTHER, G.Z., LYNCH, J.M., BETZ, S.J. & HENSON, P.N. (1980). Human neutrophil-derived platelet-activating factor. *J. Immunol.*, **124**, 676.

MING, W.J., BERSANI, L. & MANTOVANI, A. (1987). Tumor necrosis factor is chemotactic for monocytes and polymorphonuclear leukocytes. *J. Immunol.*, **138**, 1469.

MORIKAWA, K., KAMEGAYA, S., YAMAZAKI, M. & MIZUNO, D. (1985*a*). Hydrogen peroxide as a tumoricidal mediator of polymorphonuclear leukocytes induced by a linear β-l, 3-D-glucan and some other immunomodulators. *Cancer Res.*, **45**, 3482.

MORIKAWA, K. KIKUCHI, Y., ABE, S., YAMAZAKI, M. & MIZUNO, D. (1984). Early cellular responses in the peritoneal cavity of mice to antitumor immunomodulators. *Gann*, **75**, 370.

MORIKAWA, K., TAKEDA, R., YAMAZAKI, M. & MIZUNO, D. (1985*b*). Induction of tumoricidal activity of polymorphonuclear leukocytes by a linear β-l, 3-D-glucan and other immunomodulators in murine cells. *Cancer Res.*, **45**, 1496.

NATHAN, C.F., SILVERSTAIN, S.C., BRUKNER, L.H. & COHN, Z.A. (1979). Extracellular cytolysis by activated macrophages and granulocytes. II. Hydrogen peroxide as a mediator of cytotoxicity. *J. Exp. Med.*, **149**, 100.

OKUTOMI, T., NAKAJIMA, Y., SAKAKIBARA, F., KAWAUCHI, H. & YAMAZAKI, M. (1987). Induction of release of cytotoxin from murine bone marrow cells by an animal lectin. *Cancer Res.*, **47**, 47.

OLD, L.J. (1987). Tumor necrosis factor: polypeptide mediator network. *Nature*, **326**, 330.

RUBIN, B.Y., ANDERSON, S.L., SULLIVAN, S.A., WILLIAMSON, B.D., CARSWELL, E.A. & OLD, L.J. (1986). Nonhematopoietic cells selected for resistance to tumor necrosis factor produce tumor necrosis factor. *J. Exp. Med.*, **164**, 1350.

RUFF, M.R. & GIFFORD, E. (1980). Purification and physicochemical characterization of rabbit tumor necrosis factor. *J. Immunol.*, **125**, 1671.

TSUNAWAKI, S., OSHIMA, H., MIZUNO, D. & YAMAZAKI, M. (1983). Induction of polymorphonuclear leukocyte-mediated cytolysis by wheat germ agglutinin and antitumor antibody. *Gann*, **74**, 258.

YAMAZAKI, M., IKENAMI, M., TSUNAWAKI, S., KAMIYA, H., NATORI, S. & MIZUNO, D. (1983). Polymorphonuclear leukocyte-mediated cytolysis by animal lectin. *Gann*, **74**, 576.

YOSHINAGA, M., GOTO, F., GOTO, K. & 4 others (1985). Biosynthesis and purification of IL-1-like factor from polymorphonuclear leukocytes of rabbits with tumor-induced granulocytosis. In *The Physiological, Metabolic. and Immunobiological Actions of Interleukin-1*, Kluger, M.J., Oppenheim, J.J. & Ponanda, M.C. (ed) p. 385. Alan R. Liss: New York.